# Ankle arthrodesis and total ankle replacement – Variations in surgical practice across England using Hospital Episode Statistics database

**DOI:** 10.1371/journal.pone.0359847

**Published:** 2026-10-15

**Authors:** Rebecca Martin, Toby Jennison, William Keith Gray, Sarah Johnson-Lynn, Tim Briggs, David Townshend

**Affiliations:** 1 Getting It Right First Time Programme, NHS England, London, United Kingdom; 2 Trauma and Orthopaedic Department, North Tyneside General Hospital, Northumbria Healthcare NHS Foundation Trust, North Sheilds, United Kingdom; 3 Hull York Medical School, University of York, Heslington, York, United Kingdom; 4 Trauma and Orthopaedic Department, Derriford Hospital, University Hospitals Plymouth NHS Trust, Plymouth, United Kingdom; 5 Trauma and Orthopaedic Department, James Cook University Hospital, South Tees Hospitals NHS Foundation Trust, Middlesbrough, United Kingdom; 6 Trauma and Orthopaedic Department, Royal National Orthopaedic Hospital, Royal National Orthopaedic Hospitals Trust, Stanmore, London, United Kingdom; 7 Northumbria University, Newcastle Upon Tyne, United Kingdom; Upperline Health Inc., UNITED STATES OF AMERICA

## Abstract

**Background and Purpose:**

End stage ankle arthritis can be managed surgically by ankle arthrodesis or total ankle replacement. Not all surgeons perform both ankle arthrodesis and total ankle replacement. This study aims to determine the numbers of ankle arthrodesis and ankle replacements being performed throughout England and to analyse variation in provision.

**Methods:**

This is a retrospective study using Hospital Episode Statistics data from NHS England for the period April 2013 to March 2025 categorised geographically by Integrated Care Boards (ICB) and NHS England regions and over time by financial year.

**Results:**

A total of 26,331 procedures were performed for end stage ankle arthritis. There was variation between ICBs with regards to the proportion of ankle replacements and ankle arthrodesis being performed. Over the study period, most practices changed towards an increasing proportion of ankle replacements. Patients receiving ankle replacements travelled further for their operation compared to those receiving ankle arthrodesis.

**Conclusion:**

There is variation in the proportion of ankle replacements being performed compared to ankle arthrodesis for end stage ankle arthritis in each ICB and region. Patients are traveling further to receive an ankle replacement compared to an ankle arthrodesis. Further investigation is required into the potential causes of this variation and the availability of total ankle replacements to all patients across the UK. Patients should have access to all treatment options even if both operations are not performed by the consulting surgeon.

## Introduction

End stage ankle arthritis (ESAA) is a debilitating disease that can cause significant morbidity and impact on patients’ quality of life and ability to work [1]. The incidence within the United Kingdom (UK) has been estimated to be 47.7 per 100,000 [2]. The traditional treatment for ESAA has been an ankle arthrodesis (AA) and the functional outcomes and patient satisfaction from this procedure are generally good [3]. However, there are concerns about the rates of nonunion and development of later adjacent joint arthritis [4,5]. Despite widespread use within the UK, there is limited data on the number of patients that undergo an AA and their long term outcomes.

A further option for ESAA is a total ankle replacement (TAR). TAR has become more popular in the last decade with the National Joint Registry of England and Wales (NJR) demonstrating that the number of TARs has more than tripled from 402 to 1,347 per annum in the period between 2010–2024 [6]. The potential benefits of TAR over AA include retaining ankle motion and protecting adjacent joints. A concern with replacement is the risk of failure, with the need to convert to either a revision ankle replacement or to an ankle arthrodesis. However, newer implants have demonstrated improved survivorship with satisfactory outcomes and complication rates [7–9].

A large, randomised controlled trial [10] of 303 patients in the UK compared AA to TAR and found that both interventions improved Patient Reported Outcome Measure (PROM) scores in those recruited. Factors which may affect the choice of AA or TAR can include age, activity level, presence of adjacent joint arthritis, intra-articular and extra-articular deformity [11]. Decision making should be patient centred and requires an informed discussion between surgeon and patient regarding the risks and benefits of each. Not all surgeons perform ankle replacements, and the availability of both procedures may be based on geographical variation and local surgeon preference, rather than a joint decision making process between surgeon and patient.

The aim of this retrospective exploratory study was to determine the incidence of ankle arthrodesis (AA) and ankle replacements (TAR) performed in England between 2013 and 2025 year. Secondary aims were to assess geographical variation in the provision of ankle arthritis surgery and the distance patients travelled to receive their procedure.

## Methods

### Study design

This study presents an exploratory retrospective review of administrative records drawn from the Hospital Episode Statistics (HES) database for England. Managed by NHS England, the HES dataset captures information on hospital activity across NHS facilities in England. It also includes details of patients who receive treatment in independent or private hospitals when the care is funded by the NHS. However, it does not cover individuals who self-fund treatment in non-NHS hospitals. All information included within this study was in the form of anonymized data with no patient identifiable detalis.

### Ethics

Data analysis and reporting were conducted following NHS England’s current guidance for research use of Hospital Episode Statistics (HES) data. The dataset was anonymised in accordance with the ISB1523 standard governing the publication of health and social care information. Patients did not take part directly in this study, and individual consent was not required as the analysis was based on routinely collected administrative records.

### Setting

England is divided into seven regions and 42 Integrated Care Boards (ICBs) by NHS England. ICBs are responsible for meeting the healthcare needs of their catchment population. ICBs are part of the Integrated Care System (ICS) in England and these are responsible for the co-ordination and commissioning of health and care services for a specific geographical area in England [12].

### Time period and case identification

We extracted data for all patients admitted to an English hospital between 1^st^ April 2013 and 31st March 2025 undergoing either a primary TAR or AA as an elective procedure. Office of Populations Censuses and Surveys Classification of Interventions and Procedures version 4 (OPCS-4) codes were used to identify these surgical procedures within the HES dataset. Multiple potential codes can be used for both primary TAR and primary AA and so all potential codes were used to ensure data collected was as thorough as possible. For a case to be included in the study, the codes had to be present in the first position in the procedural record, indicating that the primary reason for admission to hospital was for the ankle procedure. The codes used to identify these procedures, and their descriptions, are shown in Supplementary material S1 Table.

### Inclusion and exclusion criteria

We included all patients aged 17 years and over who had an elective primary AA or primary TAR within the study period. We excluded patients who had a revision procedure, primary arthrodesis with an adjacent joint arthrodesis such as tibiotalocalcaneal arthrodesis and emergency admissions.

### Outcomes

The population of each ICB was obtained using population health information from 2024 [13] and NHS England regions population data obtained via NHS England from 2025 [14]. The numbers of primary AAs and primary TARs performed in each ICB was used to calculate the number of procedures being performed per million population as well as the proportion of each procedure performed. The proportion of procedures performed in each NHS England region was analysed per financial year to determine changing practices over time. The geographical analysis of AA and TAR prevalence was based on where the procedure was performed (provider ICB) and also where the patient’s home address was located (residence ICB). Residence ICB was determined based on Lower Super Output Area (LSOA) of residence. LSOAs are Office for National Statistics (ONS) geographical areas; a typical LSOA comprises a population of 1200–1800 people.

We identified whether patients were treated within the same ICB as their residence or a different ICB and the distance travelled from residence to treating hospital for their procedure was calculated. The distance and travel time for each patient were calculated based on the mid-point of their residence Lower Super Output Area (LSOA) and the postcode of the hospital providing treatment. Data on public transport routes and road networks across England were utilised to model theoretical journey distances. Travel distances were categorised as number of patients who travelled greater than 30 miles and number of patients who travelled greater than 50 miles.

Geographical analysis on variation could only be performed on patients who received their operation in an NHS hospital. Although HES data does capture those who have their operation in the independent sector, it does not log the location of this operation but rather the treatment providers, which typically have multiple sites across the country. Travel distance was capped at 100 miles, meaning that patients travelling further than this had a value of 100 miles recorded.

### Data management

All extracted data were stored within secure NHS England IT server space. Data were analysed within this secure environment using standard statistical software: Microsoft Excel (Microsoft Corp, Redmond, WA, USA) and Stata (Stata Corp LLC, College Station, TX, USA). All analysis reported are descriptive, using summary statistics (e.g., frequency, mean, median) as appropriate to the level of the data.

## Results

### Demographics

Between April 2013 and March 2025 there were 26,331 primary TAR or AA operations performed for ESAA in England (8,836 TARs and 17,495 AAs). For primary TAR the mean age was 67.8 years (standard deviation 10.0 years) and for primary AA the mean age was 61.0 years (standard deviation 13.6 years). A full list of patient demographic characteristics can be found in Table 1.

**Table 1:** Profile of all patients categorized as AA or TAR.

Variable	AA (N = 17,495)	TAR (N = 8,836)
**Demographic factors**		
**Age band (9 missing values)**		
17-29 years	406 (2.3%)	34 (0.4%)
30-39 years	904 (5.2%)	57 (0.6%)
40-49 years	1952 (11.2%)	259 (2.9%)
50-59 years	4134 (23.6%)	1361 (15.4%)
60-69 years	4942 (28.2%)	2963 (33.5%)
≥ 70 years	5148 (29.4%)	4162 (47.1%)
Sex (26 missing values)		
Female	5766 (33.0%)	3388 (38.4%)
Male	11719 (67.0%)	5432 (61.6%)
**Frailty**		
None	13325 (76.2%)	6747 (76.4%)
Mild	1766 (10.1%)	908 (10.3%)
Moderate	1580 (9.0%)	788 (8.9%)
Severe	824 (4.7%)	393 (4.4%)
**Deprivation (602 missing values)**		
1 (most deprived)	2656 (15.5%)	1013 (11.8%)
2	3210 (18.8%)	1391 (16.2%)
3	3671 (21.4%)	1916 (22.3%)
4	3891 (22.7%)	2076 (24.1%)
5 (least deprived)	3683 (21.5%)	2216 (25.7%)
**Length of stay (days)**		
Median	2 (IQR 1–2)	2 (IQR 1–3)
**Travel Distance (miles) (3765 missing values)**		
Range	0-100	0-100
Mean	15.99	20.63
Median	10.6	13.6
**Sector**		
NHS	14973 (85.6%)	8163 (92.4%)
Independent	2522 (14.4%)	673 (7.6%)
**Treatment location vs patient residence (excluding independent sector) (3790 missing values)**		
Treatment ICB same as residence ICB	11827 (67.6%)	6024 (68.2%)
Treatment ICB not same as residence ICB	2775 (15.9%)	1915 (21.7%)

A total of 799 consultant surgeons performed TARs and/or AAs; 21 (2.6%) performed only TAR, 472 (59.1%) performed only AA and 305 (38.2%) performed both TAR and AA.

### Procedures for ankle arthritis across the UK

There were approximately 449 operations per million population in England. The number of these procedures being performed within each NHS England region varied per million population with the lowest rate being 244.2 operations per million population and the highest rate being 589.4 operations per million population (Fig 1).

**Fig 1:**
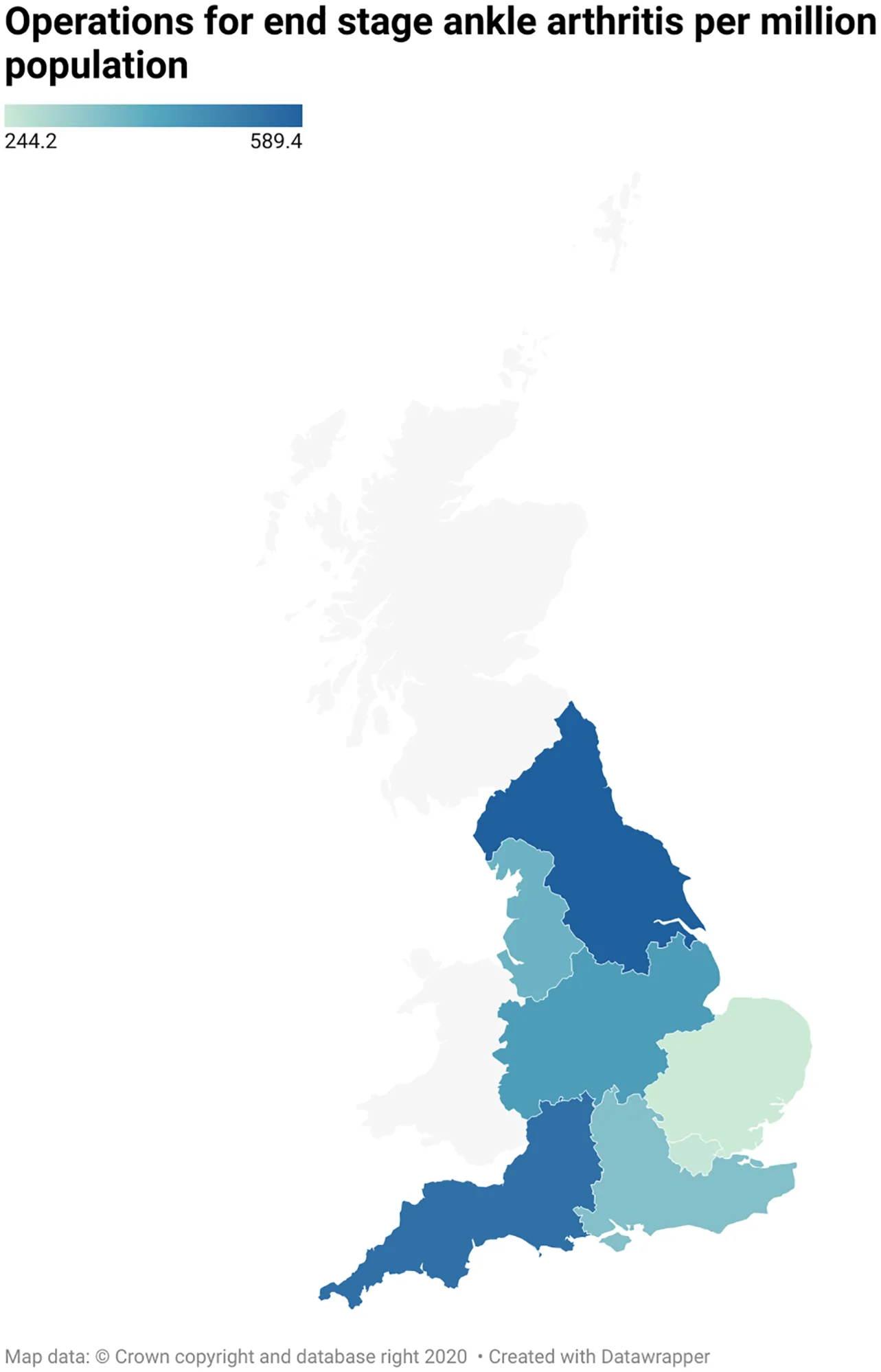
Operations for end stage ankle arthritis per million population in NHS England regions.

As shown in Fig 2, the number of AAs being performed overall showed a slight reduction with 1570 being performed in the 2013/14 financial year and 1534 being performed in the 2024/25 financial year. TARs, however, have shown an increase in use with 563 being performed in 2013/14 and 1224 being performed in 2024/25. Both procedures had a comparable reduction in numbers between 2020 and 2022 due to the COVID-19 pandemic.

**Fig 2:**
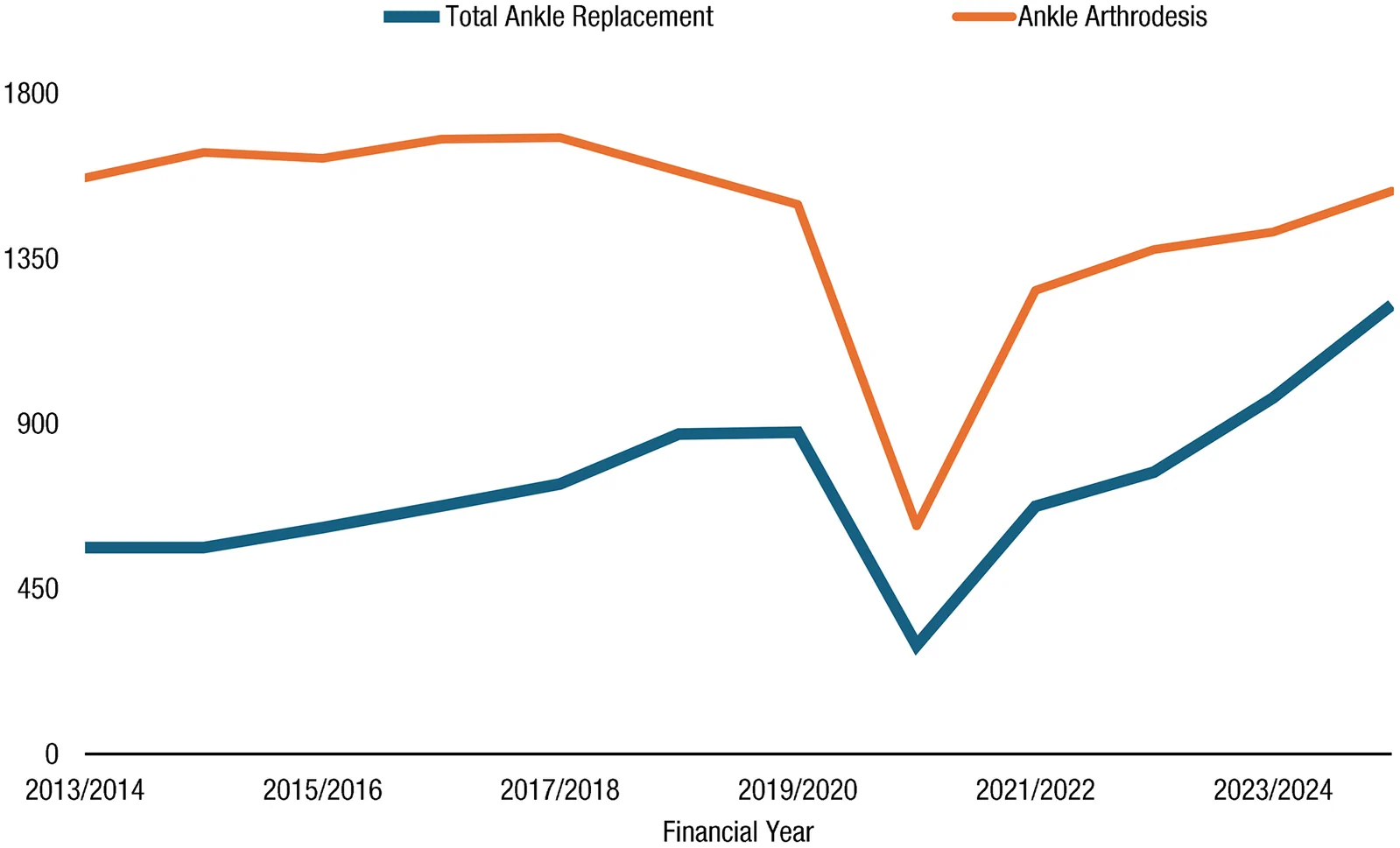
Number of ankle replacements (TAR) and ankle arthrodesis (AA) performed in each financial year.

### Variation in primary ankle arthrodesis and primary ankle replacements across the UK

There was substantial variation in the proportion of each procedure type being performed across the different ICBs. In 4 of the 42 ICBs more TARs were performed than AAs, (Fig 3). The ratio of AA:TAR ranged from 0.7:1–30:1. There was a statistically significant association between procedure type and trust ICB (χ^2^ = 2400, df = 42, p < 0.001), indicating marked geographical variation in the use of ankle replacement.

**Fig 3:**
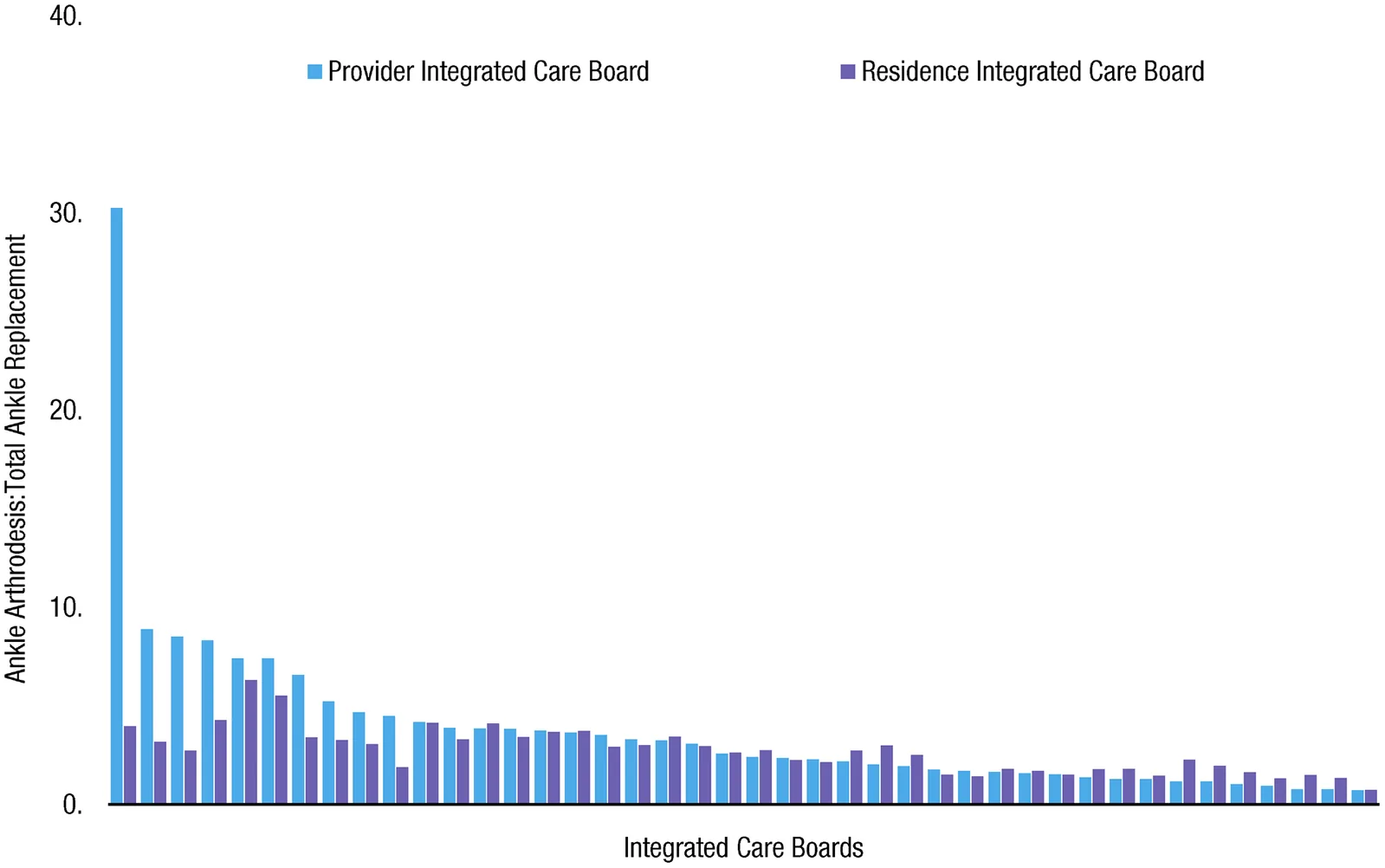
Provider Integrated care board vs residence integrated care board.

However, when considering patient residence ICB instead of trust ICB, the extent of the variation was less (Fig 3). Although most ICBs still had a predominance of patients receiving AAs, the maximum AA to TAR ratio was 6:1. Data on whether the provider ICB was different to the residence ICB are presented in Table 1. There was a higher proportion of patients travelling to an ICB outside of their residence ICB when receiving TARs compared to AAs (24.2% vs 18.0%).

### Changing practice in ankle arthritis surgery

We categorised the data into three groups of four financial years (2013–2017, 2017–2021, and 2021–2025) to investigate trends in AA and TAR provision by NHS region over time (Fig 4). There has been an overall shift to performing a higher proportions of TARs throughout the regions, with a reduction in inter-regional variation.

**Fig 4:**
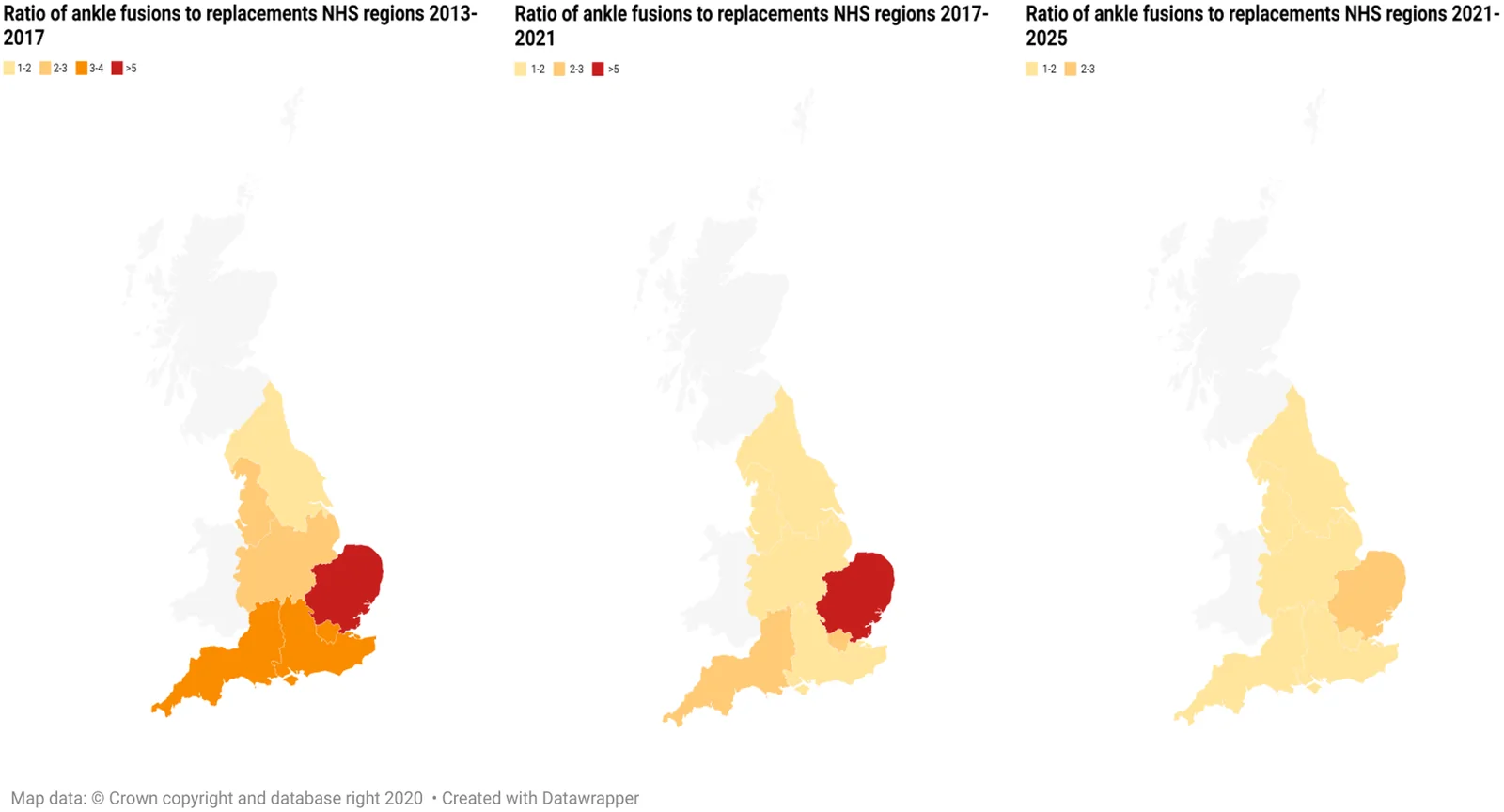
The ratio of ankle arthrodesis to replacements per NHS regions over 12 years.

### Travel distances for procedure

In total, 493 (1.9%) patients out of the 26,331 travelled further than 100 miles for surgery and had their distance capped at 100 miles Patients travelled further for a TAR (mean 20.6 miles) compared to an AA (mean 16.0 miles) (supplementary S2 Table). There was large variation between the different NHS regions in the distance travelled per operation (supplementary table 2). The furthest median distance travelled for TAR was 20 miles in the North West region and the furthest median distance travelled for AA was 14.4 miles in the East of England. The region with the highest percentage of patients travelling ≥30 miles for a TAR was the North West and the region with the highest percentage of patients travelling ≥50 miles for a TAR was the South East.

## Discussion

This study is the first to present national data on the relative incidence of AAs and TARs within England. It was demonstrated that the overall ESAA surgery rates were 449 per million population. There is, however, significant variation amongst the different ICB’s in choice of procedure.

We found that the proportion of TARs compared to AAs has increased over the 12 years of this study. This observation is supported by NJR data which shows the volume of ankle replacements increasing year on year [6]. Similar studies in the USA have also demonstrated an increase in TARs without increase in AAs [15–17]. Finland [18] and Italy [19] also report a rise in the use of TARs and a decline in the use of AA. The Finnish study covered a period from 1997 to 2018 and demonstrated an increase in TARs from 0.3 to 0.9 per 100,000 population with a decrease in AA use from 4.4 to 3.8 per 100,000 population. The Italian study covered a similar length of time from 2001 to 2022 and showed that the yearly number of TARs being performed increased from 96 per year to 996 per year. In contrast, a national database study from Germany which analysed data between 2008 and 2018 identified a decrease over time of ankle replacement use in favour of AA with TAR use declining by 39% and AA use increasing by 31% [20].

There was a difference between the distance travelled for AA and TAR, with TAR patients travelling further on average per procedure compared to AA. Shaffrey et al, when looking at a series of 160 patients have reported that patients who travel greater distances (>50 miles) for TAR compared to those who travel <50 miles do not have an increased rate of complications or worse patient reported outcome measures [21]. This is supported by a study [22] which found that increasing travel distances in the setting of complex revision knee replacement did not increase the 30 day readmission rate.

Outcomes for TAR are improving with the introduction of new implant design, improved instrumentation and improved surgical techniques [23], although there is evidence that younger patients and those with raised BMI have higher failure rates [24]. Benefits of TARs in terms of patient function, gait, range of movement and pain relief are becoming more apparent [10,25]. This is likely to make TAR an increasingly attractive option in years to come.

It is difficult and unlikely to be advisable to recommend an ideal ratio of AAs to TARs at a population level. The high degree of variation in procedure offered across ICBs and regions in England is however, likely to be unwarranted. It is highly unlikely to arise though patient choice or case mix alone. It is important that, where a patient may be suitable for either TAR or AA the benefits and limitations of each procedure should be presented to them, independent of the location of the hospital provider. Ankle arthritis networks have been recommended to mitigate against unwarranted variation in practice and facilitate onward referral to ensure equity of access to the most appropriate surgical intervention [26,27].

### Limitations

There are several limitations to this study. The first is, as with any data registry study, that there may be inaccuracies in data coding [28]. A further limitation is that there are no specific codes for ankle fusion. Therefore, whilst every effort was made to include all potential variations of codes to allow a comprehensive dataset, there are likely to be occasional procedures that were missed due to coding errors and inaccuracies. Patients captured were those who had their procedure within England and so may miss any patient who travelled to Scotland or Wales for treatment. This study was a direct comparison on rates of TAR and AA within England and did not take into consideration the severity of the ankle arthritis or the presence of deformity which could potentially influence the operation undertaken. The study focuses on geographical variation and did not take into consideration possible co-morbidities or demographics which could influence decision between operations.

## Conclusion

The proportion of TARs being performed throughout the NHS England regions has increased between 2013 and 2025. There is variation in which procedures are performed by each trust and to a lesser extent variation in which procedure patients undergo according to their area of residence within England.

Further investigation is required into the potential causes behind the variation across ICBs and regions in the availability of TARs. We recommend all treatment options for ESAA should be considered and the patients should be counselled on the options available to them in light of their presentation. Patients should have access to all treatment options even if both operations are not performed by the consulting surgeon. The fact that patients are travelling for TAR is an encouraging indicator that if ankle arthritis networks were introduced within England, patients would be willing to travel if required to receive their preferred treatment.

## Supporting information

S1 Table OPCS codes. (DOCX)

S2 Table Travel distance per procedure in NHS regions. (DOCX)
