# Supplementary material for: Ankle arthrodesis and total ankle replacement – Variations in surgical practice across England using Hospital Episode Statistics database
Source: PLoS One. 2026 Oct 15;21(10):e0359847. doi: 10.1371/journal.pone.0359847 (PMC13671524; doi:10.1371/journal.pone.0359847)
Supplement: Supplementary file 1 [file pone.0359847.s001.docx]

| Procedure  Table S1: OPCS codes | Code | Description |
| --- | --- | --- |
| Primary TAR | | |
|  | O321 | Primary total prosthetic replacement of ankle joint NEC |
|  | O328 | Other specified total prosthetic replacement of ankle joint |
|  | O329 | Unspecified total prosthetic replacement of ankle joint |
| Only if Z856 is also listed: | W431 | Primary total prosthetic replacement of joint using cement NEC |
|  | W438 | Other specified total prosthetic replacement of other joint using cement |
|  | W439 | Unspecified total prosthetic replacement of other joint using cement |
|  | W441 | Primary total prosthetic replacement of joint not using cement NEC |
|  | W448 | Other specified total prosthetic replacement of other joint not using cement |
|  | W449 | Unspecified total prosthetic replacement of other joint not using cement |
|  | W451 | Primary total prosthetic replacement of joint NEC |
|  | W458 | Other specified other total prosthetic replacement of other joint |
|  | W459 | Unspecified other total prosthetic replacement of other joint |
|  | W521 | Primary prosthetic replacement of articulation of bone using cement NEC |
|  | W528 | Other specified prosthetic replacement of articulation of other bone using cement |
|  | W529 | Unspecified prosthetic replacement of articulation of other bone using cement |
|  | W531 | Primary prosthetic replacement of articulation of bone not using cement NEC |
|  | W538 | Other specified prosthetic replacement of articulation of other bone not using cement |
|  | W539 | Unspecified prosthetic replacement of articulation of other bone not using cement |
|  | W541 | Primary prosthetic replacement of articulation of bone NEC |
|  | W548 | Other specified other prosthetic replacement of articulation of other bone |
|  | W549 | Unspecified other prosthetic replacement of articulation of other bone |
| Primary Fusion | | |
| Only if Z856 is also listed: | W601 | Primary arthrodesis and extra-articular bone graft NEC |
|  | W609 | Unspecified fusion of other joint and extra-articular bone graft |
|  | W611 | Primary arthrodesis and articular bone graft NEC |
|  | W619 | Unspecified fusion of other joint and other articular bone graft |
|  | W621 | Primary arthrodesis and internal fixation of joint NEC |
|  | W622 | Primary arthrodesis and external fixation of joint NEC |
|  | W628 | Other specified other primary fusion of other joint |
|  | W629 | Unspecified other primary fusion of other joint |
