# Supplementary material for: Ankle arthrodesis and total ankle replacement – Variations in surgical practice across England using Hospital Episode Statistics database
Source: PLoS One. 2026 Oct 15;21(10):e0359847. doi: 10.1371/journal.pone.0359847 (PMC13671524; doi:10.1371/journal.pone.0359847)
Supplement: Supplementary file 2 [file pone.0359847.s002.docx]

| Table S2: Travel distances per procedure in NHS regions | | | |
| --- | --- | --- | --- |
| **NHS Region**  **(missing = 609)** |  | **TAR** | **AA** |
| **East of England** | **Number** | 482  (missing = 39) | 1537  (missing = 168) |
|  | **Median (miles)** | 19.1 | 14.4 |
|  | **≥30 miles (% of patients)** | 28.4% | 18.8% |
|  | **≥50 miles (% of patients)** | 12.0% | 6.4% |
| **London** | **Number** | 351  (missing = 21) | 1113  (missing = 70) |
|  | **Median (miles)** | 7.6 | 6.3 |
|  | **≥30 miles (% of patients)** | 6.0% | 4.8% |
|  | **≥50 miles (% of patients)** | 4.0% | 1.9% |
| **Midlands** | **Number** | 1478  (missing = 152) | 3115  (missing = 575) |
|  | **Median (miles)** | 14.9 | 12.3 |
|  | **≥30 miles (% of patients)** | 22.7% | 14.5% |
|  | **≥50 miles (% of patients)** | 9.5% | 5.4% |
| **North East and Yorkshire** | **Number** | 2197  (missing = 235) | 2645  (missing = 422) |
|  | **Median (miles)** | 10.2 | 8.9 |
|  | **≥30 miles (% of patients)** | 19.6% | 9.6% |
|  | **≥50 miles (% of patients)** | 9.0% | 3.8% |
| **North West** | **Number** | 1227  (missing = 94) | 1735  (missing = 357) |
|  | **Median (miles)** | 20 | 8 |
|  | **≥30 miles (% of patients)** | 31.7% | 15.2% |
|  | **≥50 miles (% of patients)** | 5.5% | 4.8% |
| **South East** | **Number** | 1179  (missing = 50) | 2328  (missing = 508) |
|  | **Median (miles)** | 15.1 | 12.2 |
|  | **≥30 miles (% of patients)** | 20.6% | 16.5% |
|  | **≥50 miles (% of patients)** | 10.7% | 6.9% |
| **South West** | **Number** | 1024  (missing = 82) | 2155  (missing =384) |
|  | **Median (miles)** | 13.3 | 12.3 |
|  | **≥30 miles (% of patients)** | 22.5% | 14.2% |
|  | **≥50 miles (% of patients)** | 8.2% | 4.3% |
